# Pharmacological separation of early afterdepolarizations from arrhythmogenic substrate in ΔKPQ *Scn5a* murine hearts modelling human long QT 3 syndrome

**DOI:** 10.1111/j.1748-1716.2007.01770.x

**Published:** 2008-04

**Authors:** G Thomas, M J Killeen, A A Grace, C L-H Huang

**Affiliations:** 1 Section of Cardiovascular Biology, Department of Biochemistry, University of Cambridge Cambridge, UK; 2 Physiological Laboratory, University of Cambridge Cambridge, UK

**Keywords:** arrhythmogenesis, long QT3 syndrome, propranolol

## Abstract

**Aim:**

To perform an empirical, pharmacological, separation of early afterdepolarizations (EADs) and transmural gradients of repolarization in arrhythmogenesis in a genetically modified mouse heart modelling human long QT syndrome (LQT) 3.

**Methods:**

Left ventricular endocardial and epicardial monophasic action potentials and arrhythmogenic tendency were compared in isolated wild type (WT) and *Scn5a*+/Δ hearts perfused with 0.1 and 1 μm propranolol and paced from the right ventricular epicardium.

**Results:**

All *spontaneously beating* bradycardic *Scn5a*+/Δ hearts displayed EADs, triggered beats and ventricular tachycardia (VT; *n* = 7), events never seen in WT hearts (*n* = 5). Perfusion with 0.1 and 1 μm propranolol suppressed *all* EADs, triggered beats and episodes of VT. In contrast, triggering of VT persisted following programmed electrical stimulation in 6 of 12 (50%), one of eight (12.5%), but six of eight (75%) *Scn5a*+/Δ hearts perfused with 0, 0.1 and 1 μm propranolol respectively in parallel with corresponding alterations in repolarization gradients, reflected in action potential duration (ΔAPD_90_) values. Thus 0.1 μm propranolol reduced epicardial but not endocardial APD_90_ from 54.7 ± 1.6 to 44.0 ± 2.0 ms, restoring ΔAPD_90_ from −3.8 ± 1.6 to 3.5 ± 2.5 ms (all *n* = 5), close to WT values. However, 1 μm propranolol increased epicardial APD_90_ to 72.5 ± 1.2 ms and decreased endocardial APD_90_ from 50.9 ± 1.0 to 24.5 ± 0.3 ms, increasing ΔAPD_90_ to −48.0 ± 1.2 ms.

**Conclusion:**

These findings empirically implicate EADs in potentially initiating spontaneous arrhythmogenic phenomena and transmural repolarization gradients in the re-entrant substrate that would sustain such activity when provoked by extrasystolic activity in murine hearts modelling human LQT3 syndrome.

Sudden cardiac death (SCD) attributable to ventricular arrhythmogenesis is one of the major causes of mortality in the developed world, accounting for over 300 000 deaths per year in the USA (Kannel *et al.* 1987, Willich *et al.* 1987) and up to 70 000 deaths per year in the UK (NICE, 2000). Cardiac arrhythmias are clinically associated with a condition known as long QT syndrome, which is characterized by prolonged ventricular repolarization and a tendency to ventricular tachycardia, particularly torsades de pointes (TdP), leading to syncope and SCD. The LQT3 subtype results from gain of function mutations in the *Scn5a* gene, affecting Na^+^ channel inactivation and leading to an arrhythmogenic persistent late inward Na^+^ current (*I*_Na_) ([Bibr b48][Bibr b49]).

The induction of ventricular arrhythmias has been attributed to the following, not necessarily exclusive electrophysiological events: (1) impaired cardiac repolarization leads to small, premature depolarizations (early afterdepolarizations, EADs), interrupting the smooth repolarization phase of the action potential (AP). If EADs reach a sufficient amplitude they may initiate a premature AP (triggered beat) which may in turn precipitate into TdP (Eckardt *et al.* 1998, Killeen *et al.* 2007a). (2) Alterations in the repolarization gradients have been attributed to heterogeneous ion channel expression through the thickness of the ventricular wall. Thus, selective action potential duration (APD) prolongation or reduction within localized regions of the ventricle can alter the dispersion of repolarization; both increases (Milberg & Eckardt 2005) and decreases (Killeen *et al.* 2007a) in such ventricular transmural repolarization gradients, often quantified as the epicardial-endocardial difference in APD_90_ (ΔAPD_90_), have been associated with arrhythmogenesis at the whole heart level.

Previous isolated tissue and single cell experiments have indicated that most cardiac cell types are capable of generating EADs. However, arrhythmogenesis at the whole heart level may involve a differential expression of contrasting electrophysiological characteristics in a population of intercellularly coupled cells not detectable in single cell studies. Such intercellular coupling has been previously shown to either facilitate or suppress EAD induction (Huelsing *et al.* 2000). Furthermore, single cell studies and studies confined to isolated tissue exclude examination of important myocardial properties essential for the study of arrhythmogenesis including re-entry mechanisms and transmural gradients of repolarization. Consequently, they have failed to clarify the relationship between EADs, transmural gradients of repolarization and arrhythmogenesis at the whole heart level. The use of an intact, working myocardium for the study of arrhythmogenesis permits the measurement of multicellular parameters involving ventricular transmural gradients of repolarization and EADs, triggered beats and arrhythmias that cannot be assessed in single cell or isolated tissue preparations. The use of an intact, beating heart model thus includes all myocardial cell types and maintains intercellular coupling and thus provides more physiologically relevant information regarding the induction and propagation of arrhythmias.

We explored for empirical means of resolving the causal relationship between EADs and abnormal transmural gradients of repolarization reflected in altered ΔAPD_90_ values in genetically modified murine whole hearts modelling human long QT syndrome type 3 for the first time. This study was directly prompted by a recent report describing a similarly empirical pharmacological separation of EADs from arrhythmogenic substrate in a murine model of hypokalaemia-induced arrhythmogenesis (Killeen *et al.* 2007b). Accordingly, it made endocardial and epicardial monophasic action potential (MAP) recordings from the left ventricle of isolated, intact Langendorff-perfused ΔKPQ *Scn5a* (*Scn5a*+/Δ) and wild type (WT) hearts, exposed to a range of propranolol concentrations. Propranolol is well known to exert potent Na^+^ channel blockade occurring independently of its β-adrenoceptor blocking actions, thought important in its antiarrhythmic action (Rauls & Baker 1979, Matthews & Baker 1982). Propranolol concentrations used (0.1 and 1 μm) were based on values known to cause electrophysiological effects in clinical dose–response studies (Duff *et al.* 1983). Indeed, they are close to previous values used in earlier studies of LQTS in animal models (Shimizu & Antzelevitch 2000).

Spontaneously beating *Scn5a*+/Δ hearts following AV node ablation showed EADs and episodes of ventricular tachycardia (VT) never observed in WT hearts. Perfusion of *spontaneously* beating *Scn5a*+/Δ hearts with both 0.1 and 1 μm propranolol suppressed both EADs and episodes of VT in all hearts, and had no effect in WT hearts. In contrast, programmed electrical stimulation (PES) protocols applying premature stimuli successfully provoked VT in 50% of *Scn5a*+/Δ hearts at baseline, events never recorded from WT hearts. The lower propranolol concentrations (0.1 μm) both abolished this VT and selectively reduced APD_90_, restoring ΔAPD_90_ close to WT control values in all *Scn5a*+/Δ hearts. In contrast, at higher concentrations (1 μm), PES successfully induced VT in 75% of *Scn5a*+/Δ hearts. This proarrhythmic effect was specifically associated with a combination of a prolonged epicardial APD_90_ and reduced endocardial APD_90_, resulting in an increased ΔAPD_90_ and exacerbated arrhythmogenic substrate.

We achieve an empirical pharmacological separation of EADs from arrhythmic substrate through the empirical use of propranolol, resolving the causal relationship between EADs and arrhythmic substrate in a genetically modified murine whole heart model of an inherited human arrhythmic syndrome. These results thus (1) demonstrate the importance of *both* EADs and arrhythmic substrate in the initiation of arrhythmias in a whole heart model of arrhythmogenecity which directly corresponds to the human clinical phenotype through genetic modification of the *Scn5a* gene, (2) provide independent corroboration of such important relationships seen using alternative pharmacological strategies (Killeen *et al.* 2007b) and (3) add to recent arguments for the utility of mouse hearts in modelling human arrhythmia syndromes (Stokoe *et al.* 2007). Such a causal relationship between EADs and arrhythmic substrate as demonstrated in the present study in a genetically modified mouse heart modelling human LQT3 syndrome at the very least merits further testing.

## Methods

### Preparation of Langendorff-perfused hearts

Techniques used for the generation of *Scn5a*+/Δ mice and the preparation of whole hearts for Langendorff perfusion have been previously described (Head *et al.* 2005). Healthy, viable hearts suitable for experimentation regained a homogeneous pink coloration and spontaneous rhythmic contraction with warming. Hearts not demonstrating these features were instantly discarded prior to experimentation, to avoid false-positive results during PES (2 hearts discarded out of a total of 25). Hearts were perfused with physiological perfusion buffer for 30 min prior to experimentation, to minimize any residual effects of endogenous catecholamine release.

### Programmed electrical stimulation

Programmed electrical stimulation of the intact hearts used paired (1 mm inter-pole spacing) platinum stimulating and recording electrodes, positioned on the basal epicardial surface of the right ventricle. The pacing protocols used 2 ms square-wave stimuli with amplitudes three times excitation threshold (Grass S48 stimulator; Grass-Telefactor, Slough, UK). PES is an established method of arrhythmia provocation that has been previously used to assess arrhythmogenecity in the murine heart ([Bibr b21][Bibr b22]). All experimental mice were bred from a 129 genetic background, which, along with C57 mice, are less susceptible to PES-induced arrhythmias than FBV or Black Swiss animals (Maguire *et al.* 2003). Nevertheless, complex pacing protocols involving double/triple extra-stimuli and rapid burst pacing were avoided to reduce the risk of false-positive results (Maguire *et al.* 2003). PES protocols comprised a drive train of eight paced S1 beats at 125 ms basic cycle length (BCL), followed by premature S2 extrastimuli every ninth beat. S1S2 intervals first equalled the pacing interval and were then successively reduced by 1 ms with each nine beat cycle until ventricular refractoriness was reached, whereupon the S2 stimuli elicited no response. All PES experiments were performed in hearts in which AV node block had not been induced.

### Monophasic action potential recordings

Monophasic action potentials were recorded from the left ventricular epicardium using a spring-loaded, Ag-Cl contact (2 mm tip diameter) MAP electrode (Linton Instruments; Harvard Apparatus, Edenbridge, UK) which was positioned manually. Left ventricular endocardial recordings were obtained using a custom-built electrode, constructed from two twisted strands of Teflon-coated (0.25 mm diameter) silver wire (99.99% purity) (Advent Research Materials Ltd, Oxford, UK), galvanically chlorided and introduced into the left ventricular cavity through a small access window created in the interventricular septum and rotated such that the tip came to rest against the free wall. The endocardial electrode was initially positioned by hand, and the contact maintained by custom-designed magnetic grips positioned on a metallic platform. All recordings were performed during steady state pacing at 125 ms to negate rate-dependent differences in APD. Signals were amplified and low-pass filtered appropriately for murine recordings (0.1–300 Hz) (Gould 2400S; Gould-Nicolet Technologies, Ilford, Essex, UK) then digitized using a 1401plus analogue-to-digital converter (Cambridge Electronic Design, Cambridge, UK). All electronically stored traces were analysed using Spike II software (Cambridge Electronic Design) according to previously documented criteria of a stable baseline and triangular MAP morphology, rapid upstroke phase and a consistent amplitude (Knollmann *et al.* 2001). The point of maximum positive deflection was considered to be the point of 0% repolarization, and return to baseline the point of 100% repolarization.

### Pharmacological agents

The concentrations of propranolol used in the present experiment (0.1 and 1 μm) were based on values known to cause electrophysiological effects from corresponding human, dose–response studies (Duff *et al.* 1983), and were in keeping with previously published studies of animal models of LQTS whereby a range of concentrations have been used (0.1–3.0 μm) (Shimizu & Antzelevitch 2000). Propranolol (Sigma-Aldrich, Poole, UK) was stored at −20 °C as a 1 mm stock solution in distilled water before dilution to the final drug concentrations in physiological buffer solution.

### Statistical analysis

All values were expressed as mean ± SEM and different experimental groups compared by one-way analysis of variance using spss software. *P* < 0.05 was considered statistically significant.

## Results

Long QT syndrome type 3 (LQT3) is associated with genetic modifications of the *Scn5a* gene, which interfere with Na^+^ channel inactivation and lead to persistent, arrhythmogenic late Na^+^ currents (Head *et al.* 2005). Recent reports have described murine hearts with such a genetic modification of the cardiac Na^+^ channel: these show a prolonged repolarization phase, altered transmural gradients of repolarization and EADs. These yield arrhythmic preparations showing triggered activity and episodes of non-sustained VT that thereby fully recapitulate the human clinical phenotype of LQT3 (Head *et al.* 2005, Thomas *et al.* 2007a).

We accordingly used this murine model to explore the causal relationship between EADs, transmural gradients of repolarization and the initiation and maintenance of arrhythmias in an experimental model system that directly corresponds to the human clinical phenotype of LQT3. Previous clinical and experimental studies had implicated *both* EADs and altered transmural gradients of repolarization in the initiation of arrhythmogenesis at the whole heart level but have not yet attempted to organize these phenomena into a causal sequence (Cosio *et al.* 1991, ([Bibr b13][Bibr b14], Milberg & Eckardt 2005). The experiments recorded left ventricular epicardial and endocardial MAPs from *Scn5a*+/Δ and WT murine hearts in the absence and presence of varying concentrations of propranolol.

In keeping with earlier studies using the canine ventricular wedge preparation, we perfused all murine hearts with buffer solution for a period of 30 min in order to washout residual catecholamines that may be leaking from nerve terminals (Krishnan & Antzelevitch 1991). Propranolol has well documented nonspecific pharmacological actions, blocking Na^+^ channels in addition to its β-adrenoceptor blocking effects. Therefore propranolol would be expected to antagonize any residual adrenergic tone caused by spontaneous release of catecholamines from nerve endings in addition to blocking Na^+^ channels. Nevertheless, the role of endogenous catecholamines in isolated hearts is unclear. Indeed, the presence of endogenous catecholamines has been previously reported in the rat (Weselcouch *et al.* 1995) and canine (Simaan & Fawaz 1970) isolated hearts. However, depletion of endogenous catecholamines with reserpine did not affect coronary flow or cardiac function in both the rat and canine heart (Simaan & Fawaz 1970, Weselcouch *et al.* 1995). With this in mind we acknowledge that propranolol may antagonize residual adrenergic tone in the Langendorff-perfused murine whole heart in the present study. Nonetheless, irrespective of the mechanisms of action of propranolol in the present study, we used this agent as a pharmacological tool to *empirically* separate out EADs from the arrhythmogenic substrate in a murine model of human LQT3 syndrome for the first time.

### Inhibition of EADs and spontaneous arrhythmogenesis in intrinsically beating *Scn5a*+/Δ hearts

Following isolation, cannulation and perfusion of *Scn5a*+/Δ and WT murine hearts, left ventricular epicardial and endocardial MAPs possessing a stable waveform morphology, amplitude and duration were achieved after a 10 min equilibration period. Following this, MAP waveforms remained highly reproducible throughout all experiments. Bradycardia is a known risk factor for *spontaneous* arrhythmogenesis at the whole heart level and earlier studies have reported an increased incidence of EADs and consequent ventricular arrhythmogenesis under bradycardic conditions (Fabritz *et al.* 2003a, Killeen *et al.* 2007a). Furthermore, LQT3 patients predominantly experience arrhythmic episodes at rest, under bradycardic conditions (van den Berg *et al.* 2001, Schwartz *et al.* 2001). To investigate the effects of propranolol upon EADs and spontaneous arrhythmogenesis we therefore induced AV block through crush-ablation of the AV node, in keeping with earlier methodologies (Fabritz *et al.* 2003a).

**Figure 1:**
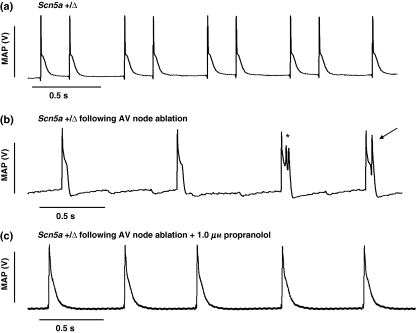
Representative monophasic action potential (MAP) recording obtained from the epicardial surface of a spontaneously-beating *Scn5a*+/Δ heart under physiological conditions (a). Following induction of complete atrioventricular (AV) block by crush ablation of the AV node, multiple early afterdepolarizations (*) are seen, along with an associated triggered action potential (arrow) (b). All such features are suppressed following perfusion with physiological buffer solution containing propranolol (1 μm) (c).

Crush ablation rendered both *Scn5a*+/Δ and WT hearts bradycardic (∼100 beats min^−1^) but nevertheless still spontaneously beating, compared to hearts in which AV block was not induced, (Fig. 1a,b). Bradycardic *Scn5a*+/Δ hearts spontaneously showed multiple EADs, triggered beats and episodes of non-sustained VT lasting on average 15 beats (range 7–33 beats) (*n* = 7) (Fig. 1b). In contrast, WT hearts never showed such events (whether EADs or polymorphic VT) following induction of AV block (*n* = 5).

We then investigated the empirical effects of a range of propranolol concentrations in such spontaneously beating bradycardic *Scn5a*+/Δ and WT hearts to explore a correlation between EADs and *spontaneous* arrhythmogenesis. Perfusion with physiological buffer containing 0.1 and 1 μm propranolol suppressed not only EADs, but also triggered beats and spontaneous VT in all seven *Scn5a*+/Δ hearts (Fig. 1c), and had no further effects upon spontaneously beating WT hearts (*n* = 5). These data therefore associate EADs with spontaneous arrhythmogenesis, and their abolition with its absence in spontaneously beating, bradycardic *Scn5a*+/Δ murine hearts. This then led to experiments that next explored the effects of propranolol upon arrhythmogenesis provoked by extrasystolic stimuli in *Scn5a*+/Δ and WT murine hearts.

### Paradoxical arrhythmogenic properties in *Scn5a*+/Δ hearts following programmed electrical stimulation

**Figure 2:**
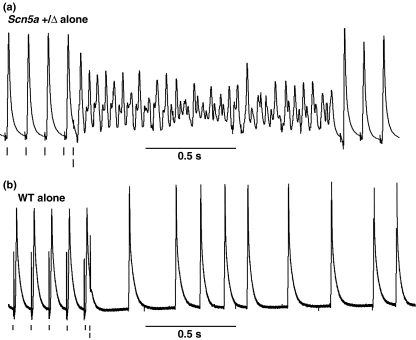
Representative trace showing monophasic action potentials recorded from the epicardial surface of *Scn5a*+/Δ and wild type (WT) hearts during programmed electrical stimulation. Following the final paced beats of the drive train at 125 ms CL (single dash), a premature stimulus (double dash) induces non-sustained polymorphic ventricular tachycardia in the *Scn5a*+/Δ (a) but not WT heart (b).

**Figure 3:**
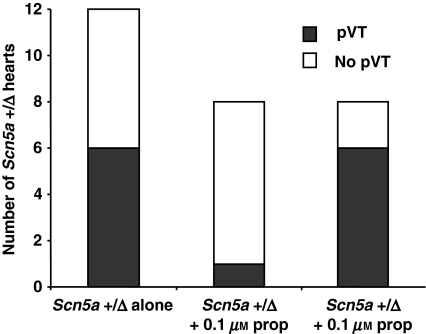
Numbers of *Scn5a*+/Δ hearts with inducible polymorphic ventricular tachycardia (pVT) during programmed electrical stimulation following perfusion with physiological buffer followed by increasing concentrations of propranolol (0, 0.1 and 1 μm).

We then investigated the effects of propranolol upon provoked arrhythmogenesis using PES that incorporated extrasystolic stimuli in *Scn5a*+/Δ and WT murine hearts. The application of PES to *Scn5a*+/Δ (*n* = 12) and WT (*n* = 10) hearts following perfusion with physiological buffer solution induced VT in 6 of 12 *Scn5a*+/Δ hearts (Fig. 2a) but not in any WT control hearts (*n* = 10) (Fig. 2b). PES was then repeated in *Scn5a*+/Δ (*n* = 8) and WT (*n* = 8) hearts at 30 min intervals, following perfusion with physiological buffer solution containing increasing concentrations of propranolol of 0.1 and 1 μm respectively. Subsequent PES procedures induced VT in one of eight *Scn5a*+/Δ hearts perfused with 0.1 μm propranolol as summarized in Figure 3. However, VT was induced in six of eight *Scn5a*+/Δ hearts following perfusion with 1 μm propranolol, confirming our previous observations (Head *et al.* 2005). In contrast, PES failed to induce VT in all WT preparations at all concentrations of propranolol.

The present PES results thus demonstrated that *low* concentrations of propranolol (0.1 μm) exerted an *antiarrhythmic* effect in *Scn5a*+/Δ hearts, findings that did correlate with the antiarrhythmic properties in spontaneously beating hearts described above. However, a higher concentration of propranolol (1 μm) reversed this correlation by exerting a *proarrhythmic* effect in *Scn5a*+/Δ hearts. This was in direct contrast to findings in the spontaneously beating *Scn5a*+/Δ hearts in which both the 0.1 and 1 μm concentrations of propranolol suppressed unprovoked arrhythmogenesis. This prompted us to explore whether these paradoxical, even if empirical, effects of propranolol in *Scn5a*+/Δ hearts might correlate with similar paradoxical alterations in the transmural gradient of repolarization. Accordingly, we investigated the effects of propranolol upon the transmural repolarization gradients known to exist within the left ventricle of WT (Anumonwo *et al.* 2001, Knollmann *et al.* 2001, Dilly *et al.* 2006) and ΔKPQ *Scn5a* mice (Thomas *et al.* 2007a). These measurements permit the quantification of the transmural gradient of repolarization, alterations which have been recently associated with arrhythmogenecity at the whole heart level ([Bibr b21][Bibr b22], Thomas *et al.* 2007a).

### The paradoxical arrhythmogenic properties correlate with opposite shifts in transmural gradients of repolarization in *Scn5a*+/Δ hearts

**Figure 4:**
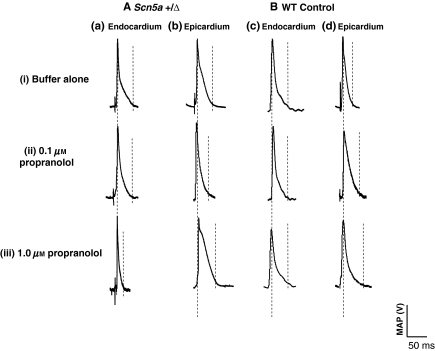
Representative traces of monophasic action potentials (MAPs) recorded from endocardial (a,c) and epicardial (b,d) surfaces of *Scn5a*+/Δ (A) and wild type (WT) (B) hearts during baseline epicardial pacing at 125 ms, following perfusion with physiological buffer solution containing increasing concentrations of propranolol (0, 0.1 and 1 μm) [(i)–(iii)]. Baseline endocardial and epicardial APD_90_ are indicated by vertical dotted lines.

**Figure 5:**
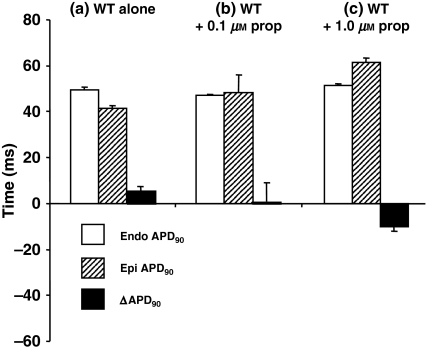
Comparison of the effect of 0, 0.1 and 1 μm propranolol on mean ± SEM endocardial APD_90_ (Endo), epicardial APD_90_ (Epi) and ΔAPD_90_ from monophasic action potential recordings in wild type (WT) hearts (*n* = 5 in each case).

**Figure 6:**
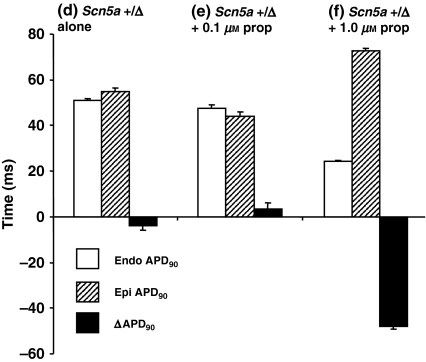
Comparison of the effect of 0, 0.1 and 1 μm propranolol on mean ± SEM endocardial APD_90_ (Endo), epicardial APD_90_ (Epi) and ΔAPD_90_ from monophasic action potential recordings in *Scn5a*+/Δ hearts (*n* = 5 in each case).

Baseline endocardial (Fig. 4a,c) and epicardial (Fig. 4b,d) APs were measured in *Scn5a*+/Δ (Fig. 4a) and WT (Fig. 4b) hearts from left ventricular MAPs recorded during steady state, right ventricular epicardial pacing at 125 ms BCL. In WT hearts, endocardial APD_90_ was significantly greater than epicardial APD_90_ (49.1 ± 1.2 vs. 42.9 ± 1.4 ms, respectively, *n* = 5, *P* < 0.05), resulting in a positive ΔAPD_90_ value of 6.2 ± 1.8 ms (Fig. 5a). In contrast, however, epicardial APD_90_ in *Scn5a*+/Δ hearts was significantly greater than the corresponding WT value (54.7 ± 1.6 ms, *n* = 5, *P* < 0.05) (Fig. 6d). Endocardial APD_90_ in *Scn5a*+/Δ hearts was calculated 50.9 ± 1.0 ms (*n* = 5) and was not statistically significant from the corresponding WT value (Fig. 5). This resulted in a negative ΔAPD_90_ value of −3.8 ± 1.6 ms in *Scn5a*+/Δ hearts.

All preparations were then perfused for 30 min each with perfusion buffer containing increasing concentrations of propranolol (0.1 and 1.0 μm) [Fig. 4(ii),(iii)]. In WT hearts, propranolol (0.1 μm) significantly prolonged epicardial APD_90_ from 42.9 ± 1.4 ms to 48.1 ± 8.0 ms (*n* = 5, *P* < 0.05), and had no significant effect upon endocardial APD_90_ (49.1 ± 1.2 vs. 47.1 ± 0.3 ms, respectively, *n* = 5). These effects resulted in a ΔAPD_90_ value of 1.0 ± 8.0 ms, not significantly different to control values (Fig. 5a,b). In *Scn5a*+/Δ hearts, perfusion with propranolol (0.1 μm) significantly reduced epicardial APD_90_ from 54.7 ± 1.6 to 44.0 ± 2.0 ms (*n* = 5, *P* < 0.05), but did not significantly alter endocardial APD_90_ values in *Scn5a*+/Δ hearts (50.9 ± 1.0 ms to 47.5 ± 1.5 ms, respectively, *n* = 5). This effect led to an appreciable increase in ΔAPD_90_ from −3.8 ± 1.6 to 3.5 ± 2.5 ms in *Scn5a*+/Δ hearts (*n* = 5) (Fig. 6).

However, increased propranolol concentrations (1.0 μm) caused progressive prolongation of epicardial APD_90_ in WT hearts to 61.6 ± 1.8 ms (*n* = 5, *P* < 0.05). In corresponding endocardial recordings, however, perfusion of WT hearts with propranolol did not significantly affect endocardial APD_90_ (51.4 ± 0.7 vs. 47.5 ± 1.5 ms, respectively, *n* = 5). These effects of 1 μm propranolol in WT hearts thus significantly reduced ΔAPD_90_ from 1.0 ± 8.0 to −10.2 ± 1.9 ms (*P* < 0.05). In contrast, perfusion of *Scn5a*+/Δ hearts with 1.0 μm propranolol significantly increased epicardial APD_90_ to 72.5 ± 1.2 ms (*n* = 5, *P* < 0.05), and significantly shortened endocardial APD_90_ to 24.5 ±0.3 ms. These marked, contrasting effects of 1 μm propranolol upon epicardial and endocardial APD_90_, significantly altered ΔAPD_90_ to −48.0 ± 1.2 ms (*n* = 5, *P* < 0.05).

These findings precisely parallel the findings from the PES studies described above. Thus the baseline ΔAPD_90_ in *Scn5a*+/Δ hearts of −3.8 ± 1.6 ms corresponded to an induction of VT by PES in 6 of 12 preparations (50% incidence of arrhythmogenesis). The restoration of ΔAPD_90_ values close to control WT values at baseline following application of 0.1 μm propranolol was reflected in the PES studies, in which premature stimuli induced VT in only one of eight preparations (12.5% incidence of arrhythmogenesis). Finally the increased arrhythmogenic substrate of ΔAPD_90_ produced by prolongation of epicardial APD_90_ and reduction in endocardial APD_90_ bringing about a significantly increased arrhythmogenic substrate reflected in ΔAPD_90_ following perfusion of *Scn5a*+/Δ hearts with 1.0 μm propranolol corresponded to the increased arrhythmic substrate suggested by the induction of VT in six of eight preparations (75% incidence of arrhythmogenesis). Collectively these findings demonstrate the pharmacological separation of EADs from altered transmural gradients of repolarization in this genetically modified murine whole heart model of arrhythmogenesis for the first time through the application of propranolol at differential concentrations.

## Discussion

We have previously engineered a murine whole heart model of arrhythmogenesis that directly corresponds to the human clinical phenotype of LQT3 through genetic modification of the gene encoding the cardiac Na^+^ channel, *Scn5a* (Head *et al.* 2005). Murine hearts heterozygous for this mutation (*Scn5a*+/Δ hearts) have prolonged epicardial APDs, altered transmural gradients of repolarization, and show EADs and episodes of VT (Nuyens *et al.* 2001, Head *et al.* 2005, Stokoe *et al.* 2007, Thomas *et al.* 2007a). The mechanisms responsible for cardiac arrhythmogenesis in LQT3 are not fully understood, although it is believed that heterogeneous AP prolongation both facilitates the development of EADs (Volders *et al.* 2000) and creates a transmural dispersion of repolarization across the ventricular wall, capable of maintaining TdP via a re-entrant mechanism (Yan *et al.* 2001, Restivo *et al.* 2004). We took advantage of this model to explore, for the first time, the causal relationship between EADs and arrhythmic substrate of transmural gradients of repolarization, through the empirical use of propranolol, in a genetically modified murine heart which fully recapitulates the arrhythmogenic human clinical phenotype.

We empirically demonstrate a paradoxical situation in which it was possible to suppress EADs and spontaneous arrhythmias on the one hand, yet exacerbate provoked arrhythmias on the other with the same empirical manoeuvre of propranolol at a concentration of 1 μm. The latter finding correlated with increased transmural gradients of repolarization, expressed as ΔAPD_90_, and translates into clinical findings in which LQT3 patients appear to derive less benefit from β-blocker therapy than other LQT syndrome patients (Priori *et al.* 2004).

First, the present study has shown, for the first time, that propranolol suppressed EADs and spontaneous arrhythmogenesis in bradycardic *Scn5a*+/Δ hearts. Crush-ablation of the AV node in *Scn5a*+/Δ hearts resulted in a bradycardic heart rate (approximately 100 beats min^−1^). At baseline, bradycardic *Scn5a*+/Δ hearts frequently displayed EADs and episodes of VT, closely correlating with earlier findings (Thomas *et al.* 2007a). Previous studies (Damiano & Rosen 1984, Zeng & Rudy 1995) have implicated the L-type Ca^2+^ channel (LTCC) as a necessary depolarizing charge carrier for EAD induction at slow stimulation rates. The mechanism for EAD induction is generally considered to involve the recovery from inactivation of LTCCs due to prolongation of APD within their critical window voltage range (January & Riddle 1989). This theory is further supported by our recent finding in which the specific LTCC blocker nifedipine suppressed EADs and arrhythmogenesis through selective inhibition of inward Ca^2+^ currents in *Scn5a*+/Δ murine hearts (Thomas *et al.* 2007a). Perfusion with 0.1 and 1 μm propranolol not only suppressed all EADs but also episodes of VT in spontaneously beating, bradycardic *Scn5a*+/Δ hearts.

Secondly, in contrast to experiments in *spontaneously* beating hearts to assess unprovoked arrhythmogenecity, both *Scn5a*+/Δ and WT hearts were subject to premature extrasystolic ventricular depolarizations using the established arrhythmia *provocation* protocol of PES. Under control conditions, premature stimuli successfully induced VT in 6 of 12 *Scn5a*+/Δ hearts (50% arrhythmia incidence) but never in WT hearts. Following subsequent perfusion with propranolol, PES only induced VT in one of eight *Scn5a*+/Δ hearts (12.5% arrhythmia incidence) at a concentration of 0.1 μm. However, following the increase in propranolol concentration to 1 μm, PES induced VT in six of eight *Scn5a*+/Δ hearts (75% arrhythmia incidence). This finding is in sharp contrast to the antiarrhythmic findings of 1 μm in spontaneously beating *Scn5a*+/Δ hearts.

Thirdly, the findings that a given manoeuvre can prevent both EADs and VT in spontaneously beating hearts yet induce VT in provoked hearts suggests that it may be exerting *additional* proarrhythmic effects that only are unmasked under arrhythmia provocation protocols. To explain differential antiarrhythmic effects in *Scn5a*+/Δ hearts exposed to propranolol we measured the corresponding epicardial and endocardial APD, thus permitting the subsequent quantification of transmural gradients of repolarization. Previously, alterations in ventricular transmural gradients of repolarization have been associated with arrhythmogenicity at the whole heart level in the setting of hypokalaemia ([Bibr b21][Bibr b22]) and in genetically modified murine hearts modelling human LQT3 (Stokoe *et al.* 2007, Thomas *et al.* 2007a) and LQT5 syndromes (Thomas *et al.* 2007b).

In the present study we measured transmural repolarization gradients under conditions of steady-state pacing. The quantification of murine APD and transmural repolarization gradients in the spontaneously beating heart is problematic. The intrinsic variability in heart rate as observed in spontaneously beating hearts can in turn alter APDs. It is well recognized that mammalian cardiac repolarization exhibits a cycle-length dependence, with shorter APDs recorded at rapid rates and longer APDs recorded at slow rates (Sabir *et al.* 2007). These effects could have important implications when determining APDs and transmural repolarization gradients. Thus APD and repolarization gradient measurements obtained from the spontaneously beating heart may not fully represent the observed experimental conditions but may instead reflect spontaneous intrinsic increases or decreases in heart rate. Therefore, in keeping with recent studies correlating murine ventricular repolarization gradients and arrhythmogenicity reported from our laboratory ([Bibr b21][Bibr b22], Stokoe *et al.* 2007, [Bibr b44][Bibr b45]) and elsewhere (Fabritz *et al.* 2003a,b 2005, London *et al.* 2007) we recorded APDs and repolarization gradients in the presence of an extrinsic pacing protocol that eliminated any intrinsic variability in heart rate which may in turn alter APD and repolarization gradients.

The present experiments showed that administration of 0.1 μm propranolol to *Scn5a*+/Δ hearts produced selective abbreviation of epicardial APD_90_, as opposed to endocardial APD_90_, thus restoring ΔAPD_90_ to positive values close to those recorded from control WT hearts. This finding is in agreement with earlier reports demonstrating selective abbreviation of epicardial over endocardial APD_90_ in response to a variety of pharmacological agents in a range of arrhythmogenic cardiac models, restoring altered transmural gradients of repolarization and exerting an antiarrhythmic effect (Aiba *et al.* 2005, Thomas *et al.* 2007b). However, perfusion of *Scn5a*+/Δ hearts with 1 μm propranolol produced significant prolongation of epicardial APD_90_ alongside significant shortening of endocardial APD_90_. These opposite effects of 1 μm propranolol on epicardial and endocardial APD90 in *Scn5a*+/Δ hearts produced a significant increase in the arrhythmogenic substrate of ΔAPD_90_, resulting in a higher incidence of provoked arrhythmogenesis. Increases in transmural repolarization gradients have been previously correlated with arrhythmogenicity in a range of cardiac models (Antzelevitch *et al.* 1998, Aiba *et al.* 2005, Milberg & Eckardt 2005, Thomas *et al.* 2007a).

Following the administration of propranolol to WT hearts, ΔAPD_90_ was reduced to −10.2 ± 1.9 ms, however, neither spontaneous nor provoked arrhythmias were recorded under these conditions. Under baseline conditions, however, LQT3 murine hearts displayed a significantly less negative ΔAPD_90_ of −3.8 ± 1.6 ms (*P* < 0.05) alongside a 50% incidence of arrhythmogenesis. These data suggest that the ΔKPQ Scn5a murine model of human LQT3 syndrome is significantly more sensitive to changes in the transmural repolarization gradient which may precipitate arrhythmogenesis compared to WT hearts. To the best of our knowledge, this finding has not been previously reported in the setting of LQT3 syndrome.

These findings in the present study correlate well with earlier reports suggesting differential epicardial and endocardial effects of propranolol alongside an increase in arrhythmogenicity. Firstly, a study in the canine ventricular wedge preparation demonstrated that propranolol increased epicardial APD, yet shortened endocardial APD (Krishnan & Antzelevitch 1991). Such differential effects were also observed in canine tissue under conditions of simulated ischaemia (Lukas & Antzelevitch 1993). Electrophysiologically, endocardial and epicardial ventricular regions are primarily characterized by their relative densities of *I*_to_: epicardial myocytes have been reported to have a significantly higher density of *I*_to_ in the murine heart (Killeen *et al.* 2007a). These differing densities of *I*_to_ are in part responsible for the shorter APD in epicardial compared to endocardial regions (Killeen *et al.* 2007a).

Blockade of Na^+^ channels with propranolol would be expected to reduce the amplitude of phase 0. Additionally, blockade of the Na^+^ window/late Na^+^ current by propranolol would act to shorten endocardial repolarization times. Reductions in epicardial *I*_Na_ and *I*_Ca_, due to a direct effect of propranolol on the Na^+^ channel and indirect effects due to a reduction in the amplitude of phase 0, respectively, would be considerably overwhelmed by the prominent epicardial *I*_to_ component, which would open at more negative potentials. In keeping with the study in the canine ventricular wedge preparation, we documented a prolongation of epicardial APD in response to propranolol administration (Krishnan & Antzelevitch 1991).

*I*_to_ would bring the membrane potential to a more negative potential which would in turn affect *I*_Ca_. Hyperpolarization would be expected to shift the membrane potential outside the activation range of *I*_Ca_, which would in turn reduce *I*_Ca_. However, if the Ca^2+^ channels are already activated, these would be expected to carry more inward Ca^2+^ current as hyperpolarization would increase the electrical gradient acting on the Ca^2+^ ions. Inactivation of *I*_to_ would reduce outward current and would give rise to a net inward current, although *I*_Ca_ may be small and slow in onset. This delay in the onset of *I*_Ca_ would in turn delay the opening of other murine delayed rectifier repolarizing components such as *I*_Kslow_, causing prolongation of epicardial APD. Such a scheme of events has been previously proposed to take place in the canine myocardium and account for the contrasting effects of propranolol on epicardial and endocardial APD (Krishnan & Antzelevitch 1991).

In the canine wedge preparation administration of propranolol prolonged epicardial APD, however, when a higher concentration of propranolol was used a shortening effect upon epicardial APD was observed. Higher concentrations of propranolol reduced phase 0 amplitude to a critical level as to permit premature repolarization due to an overwhelming *I*_to_ (Krishnan & Antzelevitch 1991). Premature epicardial repolarization achieved through significant reductions in the amplitude of phase 0 is primarily responsible for the appearance of the epicardial extrasystole and the subsequent phase 2 re-entry mechanism which is thought to underlie arrhythmogenesis in the Brugada syndrome (Antzelevtich & Yan 2000). We did not increase propranolol concentrations beyond 1 μm in the present study due to the fact that plasma concentrations of propranolol beyond 1 μm are not clinically relevant and are therefore not likely to occur in the treatment of LQT3 syndrome patients. Higher concentrations of propranolol may have reduced phase 0 amplitude to an even greater extent in the LQT 3 murine heart which would lead to premature epicardial repolarization, in keeping with previous findings performed in the canine ventricular wedge model (Krishnan & Antzelevitch 1991).

We have previously correlated alterations in the transmural repolarization gradient with arrhythmogenicity in murine models of LQT3 syndrome (Thomas *et al.* 2007a), LQT5 syndrome (Thomas *et al.* 2007b) and hypokalaemia-induced arrhythmogenesis ([Bibr b21][Bibr b22]). However, we fully acknowledge that additional factors beyond *transmural* repolarization gradients may indeed account for the observed incidences of arrhythmias in LQT3 mouse hearts in the present study. In mammalian hearts, cardiac repolarization follows tightly regulated patterns, from epicardium to endocardium and from apex to base, which are considered to maintain cardiac pump function and to provide a safeguard against arrhythmias. In addition to transmural repolarization gradients, studies have suggested that apico-basal repolarization gradients also play an important role in arrhythmia induction. In a recent study, London *et al.* (2007) measured apex to base repolarization gradients in genetically modified murine hearts harbouring deletions of components for *I*_to_. Increases in the apex to base repolarization gradient were associated with arrhythmogenesis. Alterations in this gradient can lead to disrupted patterns of depolarization and repolarization, which may in turn lead to the development of regions of conduction block which can facilitate arrhythmogenesis through re-entrant mechanisms (Killeen & Sabir 2007). Such alterations in depolarization and repolarization patterns in an alternative mouse model of LQT3 syndrome have been recently reported (Tian *et al.* 2007). We therefore cannot exclude the fact that similar mechanisms may be taking place in the present study, which may also account for the incidences of arrhythmogenesis observed in LQT3 mice. Nevertheless, these findings in the present study demonstrating alterations in the *transmural* repolarization gradient are in agreement with recent findings describing alterations in *apico-basal* repolarization patterns in a genetically modified murine model of human LQT3 syndrome (Tian *et al.* 2007).

Measuring cardiac repolarization through MAP recordings helps to quantify changes in AP shape and duration that may correspond to an arrhythmogenic phenotype. In the present study, continuous recording of epicardial and endocardial APs in the isolated mouse heart enabled us to monitor changes in transmural gradients of repolarization throughout the duration of experiments and to correlate these changes with an altered arrhythmic tendency. Similarly, optical mapping of the isolated heart can provide important information regarding dispersion of repolarization alongside activation and repolarization patterns in the murine ventricle at a high resolution (Baker *et al.* 2000). However, optical mapping studies in the intact heart are limited to recording signals from only the epicardial surface. MAPs can be recorded continuously and simultaneously from multiple sites in the isolated heart ([Bibr b13][Bibr b14], Killeen *et al.* 2007a). In the present study, recording from ventricular epicardial and endocardial sites served as an accurate and sensitive marker for changes in transmural repolarization gradients in the mouse heart and as a useful indicator of proarrhythmia.

At the whole heart level we have previously shown that 1 μm propranolol increases electrogram duration (EGD) and increases the dispersion of ventricular conduction curves as measured through paced electrogram fractionation analysis, when administered to *Scn5a*+/Δ hearts (Head *et al.* 2005). These latter effects were also accompanied by an increase in arrhythmogenecity (Head *et al.* 2005). Increases in EGD have been previously associated with increased re-entrant substrate and a higher incidence of arrhythmogenesis at the clinical (Saumarez *et al.* 1992) and experimental (Balasubramaniam *et al.* 2003) levels.

The aim of this study was to use propranolol as an empirical means of separating conditions known to contribute to arrhythmogenesis rather than to elucidate the anti and pro-arrhythmic mechanisms of action of propranolol in *Scn5a*+/Δ hearts. Nevertheless, it has been known for some time that the antiarrhythmic effects of propranolol may be unrelated to antagonism of the β-adrenoreceptor (Woosley *et al.* 1979). Additional local anaesthetic actions of propranolol, Na^+^ channel blockade, leading to reductions in APD have previously been observed in cardiac tissue (Davis & Temte 1968, Pruett *et al.* 1980, Matthews & Baker 1982). Indeed, Matthews & Baker (1982) concluded that concentrations of propranolol required to produce a 50% blockade of Na^+^ channels closely correlated with concentrations required to exert antiarrhythmic effects on isolated rabbit atria (Rauls & Baker 1979). Additionally, at the clinical level, Duff *et al.* (1983) reported that suppression of ventricular arrhythmias in 40% of patients necessitates a plasma propranolol concentration in excess of that producing substantial β-receptor blockade. At high plasma concentrations (472 ± 68 ng ml^−1^ ≈ 1.82 μm), additional shortening of the endocardial MAP duration and QTc interval was observed, *beyond* that seen at low plasma propranolol concentrations (104 ± 17 ng ml^−1^ ≈ 0.57 μm) (Duff *et al.* 1983). These findings suggest that propranolol possesses an intrinsic efficacy of potent Na^+^ channel blockade, irrespective of its β-adrenoceptor blocking actions.

Previous reports have attempted to examine the effects of β-adrenergic stimulation and antagonism in LQT3 syndrome (Shimizu & Antzelevitch 2000, Head *et al.* 2005). Administration of isoproterenol to a surrogate, pharmacological model of LQT3 syndrome in the canine ventricular wedge preparation reduces transmural repolarization gradients through preferential abbreviation of the M-cell APD and reduces the incidence of arrhythmogenesis (Shimizu & Antzelevitch 2000). Propranolol appears to antagonize this protective effect of isoproterenol and confers a higher degree of arrhythmogenesis in the wedge preparation (Shimizu & Antzelevitch 2000). These results suggest that β-adrenoceptor stimulation may be antiarrhythmic in the setting of LQT3 syndrome. However, results from our previous study suggest otherwise: perfusion of LQT3 mice with identical isoproterenol concentrations used in the above study did not exert any antiarrhythmic effects (Head *et al.* 2005). In keeping with earlier results in the canine wedge ventricular preparation (Shimizu & Antzelevitch 2000) and clinical findings which suggest that LQT3 patients derive less benefit from β-adrenoceptor blockade therapy than other LQTS patients (Priori *et al.* 2004), administration of propranolol did not exert an antiarrhythmic effect in the LQT3 murine heart (Head *et al.* 2005). These results are in keeping with the present study in which propranolol exerted proarrhythmic effects in LQT3 murine hearts through amplification of left ventricular transmural repolarization gradients.

In conclusion, we report, for the first time, the empirical separation of EADs from arrhythmic substrate in a genetically modified mouse model of human LQT3 syndrome. In this study we used propranolol at different concentrations as a pharmacological tool to separate EADs from arrhythmic substrate of ΔAPD_90_ in an intact genetically modified whole heart model of arrhythmogenesis that models human LQT3 syndrome. Regardless of the underlying mechanism, we have demonstrated that low and high concentrations of propranolol reduced arrhythmogenecity in spontaneously beating *Scn5a*+/Δ hearts by removing the trigger for the arrhythmia, the EAD. However, high concentrations of propranolol exacerbated transmural gradients of repolarization in *Scn5a*+/Δ hearts, which resulted in a proarrhythmic effect unmasked in provoked arrhythmogenesis studies in which premature stimuli, acting as surrogate EADs, imposed upon an arrhythmic substrate.

We also show, for the first time, that higher concentrations of propranolol can exert a proarrhythmic effect in a genetically modified intact murine model of LQT3 syndrome. These findings correspond with clinical observations in which β-adrenoceptor blocker therapy has proved considerably less effective in reducing cardiac events in LQT3 patients than in the other LQTS subtypes (Moss 1998, Moss *et al.* 2000, van den Berg *et al.* 2001, Schwartz *et al.* 2001, Priori *et al.* 2004).
